# Supplementary material for: Mass Spectrometry as a Highly Sensitive Method for Specific Circulating Tumor DNA Analysis in NSCLC: A Comparison Study
Source: Cancers (Basel). 2020 Oct 16;12(10):3002. doi: 10.3390/cancers12103002 (PMC7602843; doi:10.3390/cancers12103002)
Supplement: Supplementary file 1 [file cancers-12-03002-s001.pdf]

Supplementary Materials

Mass Spectrometry as a Highly Sensitive Method for Specific Circulating Tumor DNA Analysis in NSCLC: A Comparison Study

Pierre-Jean Lamy, Paul van der Leest, Nicolas Lozano, Catherine Becht, Frédérique Duboeuf, Harry J.M. Groen, Werner Hilgers, Nicolas Pourel, Naomi Rifaela, Ed Schuuring and Catherine Alix-Panabières

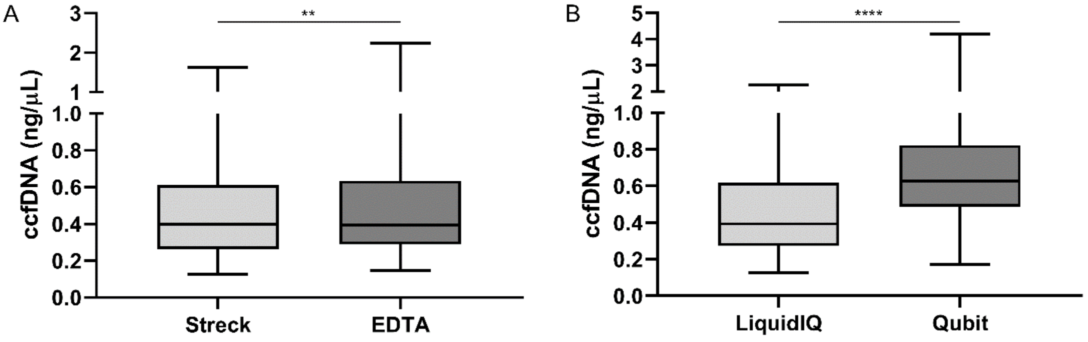

Figure S1. Comparison of ccfDNA quantitative analysis on LiquidIQ™ validation samples. (**A**) Boxplots illustrating the concentration of ccfDNA from either Streck and EDTA blood collection tubes as measured by LiquidIQ™. (**B**) Boxplots illustrating the concentration of ccfDNA from both Streck and EDTA blood collection tubes as measured by LiquidIQ™ and Qubit™. Data is presented as median (horizontal line) and interquartile range (boxes). Wilcoxon matched-pairs signed rank test, ***p* < 0.01, *****p* < 0.0001.

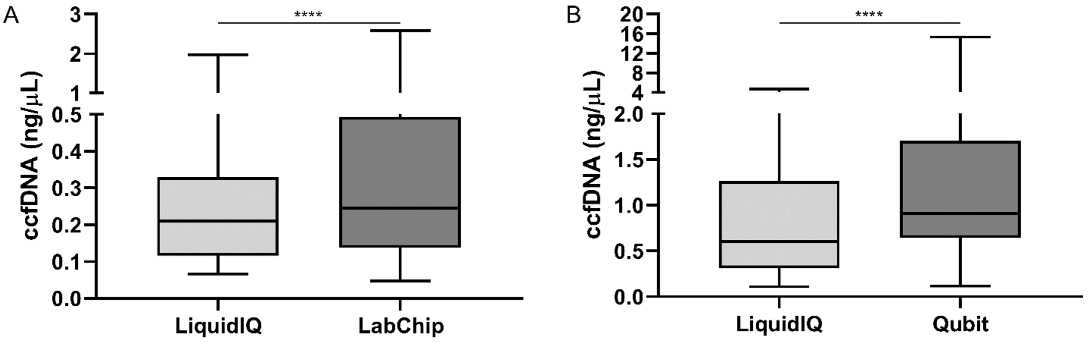

Figure S2. Methodological comparison of ccfDNA yield in MTP and GRO cohorts. (**A**) Boxplots illustrating the concentration of ccfDNA from the MTP cohort as measured by LiquidIQ™ and LabChip®. (**B**) Boxplots illustrating the concentration of ccfDNA from the GRO cohort as measured by LiquidIQ™ and Qubit™. Data is presented as median (horizontal line) and interquartile range (boxes). Results are only included when both tests were performed and evaluable. Wilcoxon matched-pairs signed rank test, *****p* < 0.0001.

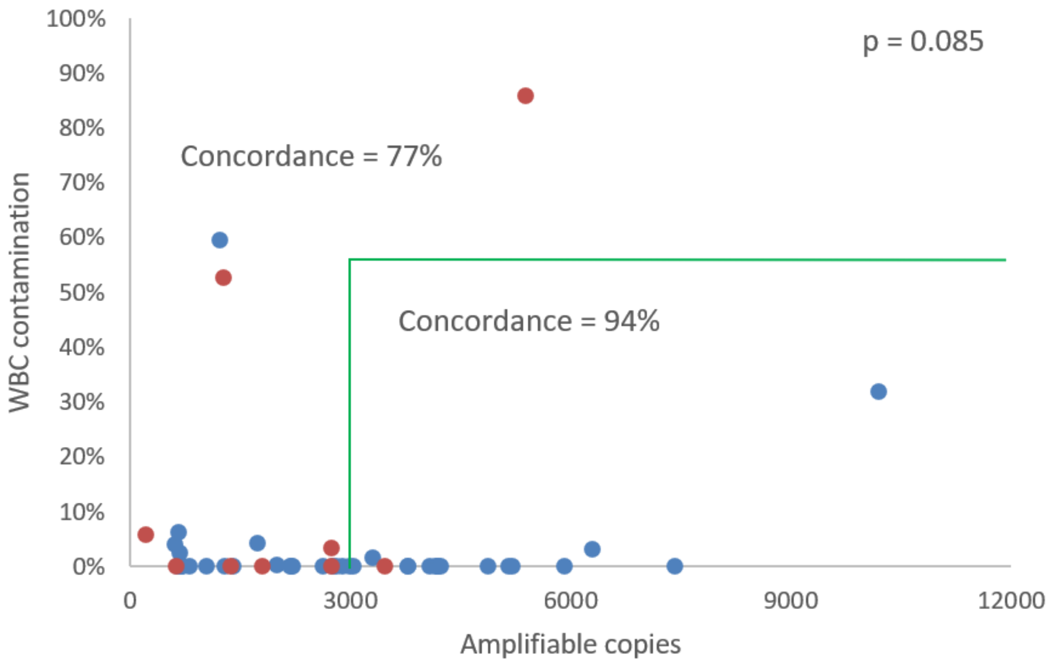

Figure S3. Correlation between UltraSEEK™ and tissue *EGFR* mutation detection in respect to Liquid IQ™ preanalytical quality control. Concordance between UltraSEEK™ ccfDNA mutation detection and tissue mutation detection is indicated by color (blue = concordant, red = discordant). Concordance is 94% when the sample contains >3000 amplifiable copies (equals 10 ng of DNA) and <50% WBC contamination.

Table S1. Mutations detectable on the UltraSEEK™ and Cobas® panels.

Gene	CDS Mutation	Amino Acid Change	COSMIC ID	UltraSEEK	Cobas
EGFR	c.2125G>A	p.E709K	12988	X	
	c.2126A>C	p.E709A	13427	X	
	c.2126A>G	p.E709G	13009	X	
	c.2126A>T	p.E709V	12371	X	
	c.2155G>T	p.G719C	6253	X	X
	c.2155G>A	p.G719S	6252	X	X
	c.2156G>C	p.G719A	6239	X	X
	c.2233_2247del(15)AAGGAATTAAGAGAA	p.K745_E749del	26038	X	X
	c.2235_2249del(15)GGAATTAAGAGAAGC	p.E746_A750del	6223	X	X
	c.2235_2248>AATTC	p.E746_A750>IP	13550	X	X
	c.2235_2252>AAT	p.E746_T751>I	13551		X
	c.2235_2251>AATTC	p.E746_T751>IP	13552	X	X
	c.2235_2255>AAT	p.E746_S752>I	12385		X
	c.2236_2250del(15)GAATTAAGAGAAGCA	p.E746_A750del	6225	X	X
	c.2236_2253del(18)GAATTAAGAGAAGCAACA	p.E746_T751del	12728	X	X
	c.2237_2251del(15)AATTAAGAGAAGCAA	p.E746_T751>A	12678	X	X
	c.2237_2254del(18)AATTAAGAGAAGCAACAT	p.E746_S752>A	12367	X	X
	c.2237_2252>T	p.E746_T751>V	12386	X	X
	c.2237_2253>TTGCT	p.E746_T751>VA	12416	X	X
	c.2237_2253>TTCCT	p.E746_T751>VP	52935	X	
	c.2237_2257>TCT	p.E746_P753>VS	18427	X	X
	c.2237_2255>T	p.E746_S752>V	12384	X	X
	c.2238_2255del(18)ATTAAGAGAAGCAACATC	p.E746_S752>D	6220	X	X
	c.2238_2248>GC	p.L747_A750>P	12422	X	X
	c.2238_2252>GCA	p.L747_T751>Q	12419	X	X
	c.2238_2252del(15)TAAGAGAAGCAACAT	p.L747_T751del	23571		X
	c.2239_2247del(9)TTAAGAGAA	p.L747_E749del	6218	X	X
	c.2239_2253del(15)TTAAGAGAAGCAACA	p.L747_T751del	6254		X
	c.2239_2256del(18)TTAAGAGAAGCAACATCT	p.L747_S752del	6255	X	X
	c.2239_2256>CAA	p.L747_S752>Q	12403	X	X
EGFR	c.2239_2248TTAAGAGAAG>C	p.L747_A750>P	12382	X	X
	c.2239_2251TTAAGAGAAGCAA>C	p.L747_T751>P	12383	X	X
	c.2239_2258>CA	p.L747_P753>Q	12387	X	X
	c.2240_2251del(12)TAAGAGAAGCAA	p.L747_T751>S	6210	X	X

	c.2240_2254del(15)TAAGAGAAGCAACAT	p.L747_T751del	12369	X	X
	c.2240_2257del(18)TAAGAGAAGCAACATCTC	p.L747_P753>S	12370	X	X
	C.2253_2276del(24)CATCTCCGAAAGCCAACAAGGAAA	p.S752_I759del	13556		X
	c.2303G>T	p.S768I	6241	X	X
	c.2307_2308ins(9)GCCAGCGTG	p.V769_D770insASV	12376	X	X
	c.2308_2309ins(9)CCAGCGTGG	p.V769_D770insASV	12426	X	
	c.2309_2310AC>CCAGCGTGGAT	p.V769_D770insASV	13558	X	X
	c.2310_2311insGGT	p.D770-N771insG	12378	X	X
	c.2311_2312ins(9)GCGTGGACA	p.D770_N771insSVD	13428	X	X
	c.2319_2320ins(9)AACCCCCAC	p.H773_V774insNPH	12381	X	
	c.2319_2320InsCAC	p.H773-V774insH	12377	X	X
	c.2369C>T	p.T790M	6240	X	X
	c.2389T>A	p.C797S	N/A	X	
	c.2390G>C	p.C797S	5945664	X	
	c.2573T>G	p.L858R	6224	X	X
	c.2573_2574TG>GT	p.L858R	12429		X
	c.2582T>A	p.L861Q	6213	X	X
	c.2582T>G	p.L861R	12374	X	
BRAF	c.1406G>C	p.G469A	460	X	
	c.1406G>T	p.G469V	459	X	
	c.1781A>G	p.D594G	467	X	
	c.1799T>A	p.V600E	476	X	
ERBB2	c.2324_2325ins(12)ATACGTGATGGC	p.A775_G776insYVMA	20959	X	
	c.2325_2326ins(12)TACGTGATGGCT	p.A775_G776insYVMA	12558	X	
	c.2326_2327ins(3)TGT	p.G776>VC	12553	X	
	c.2326_2327ins(3)TTT	p.G776>VC	12552	X	
KRAS	c.34G>A	p.G12S	517	X	
	c.34G>C	p.G12R	518	X	
	c.34G>T	p.G12C	516	X	
KRAS	c.35G>A	p.G12D	521	X	
	c.35G>C	p.G12A	522	X	
	c.35G>T	p.G12V	520	X	
	c.37G>T	p.G13C	527	X	
	c.38G>A	p.G13D	532	X	
	c.183A>C	p.Q61H	554	X	
	c.183A>T	p.Q61H	555	X	

	c.181C>A	p.Q61K	549	X
	c.181C>G	p.Q61E	550	X
	c.182A>C	p.Q61P	551	X
	c.182A>G	p.Q61R	552	X
	c.182A>T	p.Q61L	553	X
PIK3CA	c.1624G>A	p.E542K	760	X
	c.1633G>A	p.E545K	763	X
	c.3140A>G	p.H1047R	775	X
	c.3140A>T	p.H1047L	776	X

Table S2. Concordance of detected mutations between UltraSEEK™ and the diagnostic Cobas® test.

Sample	Liquid IQ Results		Cobas Mutations		UltraSEEK Mutations			Concordance		EGFR Tissue Mutations		
	Input (ng)	WBC Contamination	Mutation 1	Mutation 2	Mutation 1	Mutation 2	Mutation 3	Detectable on Both Panels	Clinically Relevant Mutations	Mutation 1	Mutation 2	Mutation 3
MTP-001	2.8	0%	EGFR exon19 del		EGFR exon19 del p.L474_P753>S			Concordant	Concordant	EGFR exon19 del		
MTP-002	13.2	0%	EGFR L858R		EGFR L858R			Concordant	Concordant	N/A		
MTP-003	10.8	0%	EGFR L858R		EGFR L858R			Concordant	Concordant	EGFR L858R		
MTP-004	9.6	0%	EGFR L858R		EGFR L858R			Concordant	Concordant	N/A		
MTP-005	3.1	0%	EGFR G719X		EGFR G719A	EGFR L861Q/R*		Concordant	USK detected more mutations	EGFR G719X	EGFR L861R	
MTP-006	4.9	0%	EGFR L858R		EGFR L858R			Concordant	Concordant	N/A		
MTP-007	9.3	0%	EGFR exon19 del		EGFR exon19 del p.L474_A750>P			Concordant	Concordant	EGFR exon19 del		
MTP-008	24.4	10%	EGFR L858R		EGFR L858R			Concordant	Concordant	N/A		
MTP-009	5.6	0%	EGFR L858R		WT			Cobas detected more mutations	Cobas detected more mutations	N/A		
MTP-010	4.8	0%	EGFR exon19 del		EGFR exon19 del E746_A750del			Concordant	Concordant	EGFR exon19 del		
MTP-011	9.8	0%	EGFR L858R		EGFR L858R			Concordant	Concordant	WT		
MTP-012	6.9	0%	EGFR		EGFR			Concordant	Concordant	N/A		

			L858R		L858R					
MTP-013	3.0	6%	EGFR exon19 del		WT		Cobas detected more mutations	Cobas detected more mutations	EGFR exon19 del	
MTP-014	12.2	6%	EGFR L858R		EGFR L858R		Concordant	Concordant	WT	
MTP-015	9.6	0%	EGFR exon19 del		EGFR exon19 del E746_A750del		Concordant	Concordant	N/A	
MTP-016	N/A	N/A	EGFR exon19 del		EGFR exon19 del E746_A750del		Concordant	Concordant	EGFR exon19 del	
MTP-017	10.8	0%	EGFR exon19 del		EGFR exon19 del E746_A750del		Concordant	Concordant	N/A	
MTP-018	14.3	0%	EGFR exon19 del	EGFR T790M	EGFR exon19 del E746_A750del	EGFR T790M	Concordant	Concordant	N/A	
MTP-019	6.8	0%	EGFR exon19 del	EGFR T790M	EGFR exon19 del E746_A750del	EGFR T790M	Concordant	Concordant	EGFR exon19 del	
MTP-020	9.2	0%	EGFR exon19 del	EGFR T790M	EGFR exon19 del p.E746_S752>V	EGFR T790M	Concordant	Concordant	EGFR exon19 del	
MTP-021	6.1	0%	EGFR exon19 del		WT		Cobas detected more mutations	Cobas detected more mutations	EGFR exon19 del	
MTP-022	6.2	0%	EGFR exon19 del		EGFR exon19 del p.E746_S752>V	EGFR T790M	USK detected more mutations	USK detected more mutations	EGFR exon19 del	
MTP-023	6.6	0%	EGFR L858R	EGFR T790M	EGFR L858R	EGFR T790M	Concordant	Concordant	EGFR L858R	
MTP-024	4.9	0%	EGFR exon19 del		EGFR exon19 del p.L747_A750>P		Concordant	Concordant	EGFR exon19 del	
MTP-025	11.5	0%	EGFR L858R		EGFR L858R		Concordant	Concordant	EGFR L858R	
MTP-026	14.0	3%	EGFR exon19 del	EGFR T790M	EGFR exon19 del E746_A750del	EGFR T790M	Concordant	Concordant	EGFR exon19 del	
MTP-027	4.9	0%	EGFR G719X	EGFR S768I	EGFR G719C	EGFR S768I	Concordant	Concordant	EGFR G719C	EGFR S768I
MTP-028	6.1	0%	EGFR exon19 del		EGFR exon19 del p.E746_S752>V	EGFR T790M	USK detected more mutations	USK detected more mutations	EGFR exon19 del	
MTP-029	3.1	0%	EGFR exon19 del		WT		Cobas detected more mutations	Cobas detected more mutations	EGFR exon19 del	
MTP-030	22.7	32%	EGFR	EGFR	EGFR	EGFR	Concordant	Concordant	EGFR	

			L861Q	T790M	L861Q	T790M			L861Q	
MTP-031	13.1	0%	EGFR exon19 del	EGFR T790M	EGFR exon19 del E746_A750del	EGFR T790M	Concordant	Concordant	EGFR exon19 del	
MTP-032	11.6	0%	EGFR exon19 del	EGFR T790M	EGFR exon19 del E746_A750del	EGFR T790M	Concordant	Concordant	EGFR exon19 del	
MTP-033	3.6	0%	EGFR exon19 del	EGFR T790M	EGFR exon19 del E746_A750del	EGFR T790M	Concordant	Concordant	EGFR exon19 del	EGFR T790M
MTP-034	4.4	0%	EGFR L858R		EGFR L858R		Concordant	Concordant	EGFR L858R	
MTP-035	22.0	0%	EGFR L858R		EGFR L858R		Concordant	Concordant	N/A	
MTP-036	10.7	0%	EGFR exon19 del		EGFR exon19 del E746_A750del		Concordant	Concordant	N/A	
MTP-037	17.5	0%	EGFR L858R		EGFR L858R		Concordant	Concordant	EGFR L858R	
MTP-038	9.3	0%	EGFR L858R		EGFR L858R		Concordant	Concordant	EGFR L858R	
MTP-039	6.9	1%	EGFR L861Q		EGFR L861Q		Concordant	Concordant	N/A	
MTP-040	N/A	N/A	EGFR exon19 del	EGFR T790M	-	EGFR T790M	Cobas detected more mutations	Cobas detected more mutations	N/A	
MTP-041	4.3	0%	EGFR exon19 del		EGFR exon19 del p.L747_P753>S		Concordant	Concordant	N/A	
MTP-042	7.3	2%	EGFR exon19 del	EGFR T790M	EGFR exon19 del E746_A750del	EGFR T790M	Concordant	Concordant	EGFR exon19 del	
MTP-043	8.4	0%	EGFR exon19 del		EGFR exon19 del p.L747_A750>P		Concordant	Concordant	EGFR exon19 del	
MTP-044	N/A	N/A	EGFR exon19 del	EGFR T790M	EGFR exon19 del p.L747_P753>S	EGFR T790M	Concordant	Concordant	EGFR exon19 del	
MTP-045	3.9	5%	EGFR exon19 del	EGFR T790M	EGFR exon19 del E746_A750del	EGFR T790M	Concordant	Concordant	EGFR exon19 del	
MTP-046	9.1	0%	EGFR exon19 del		EGFR exon19 del p.L747_A750>P		Concordant	Concordant	EGFR exon19 del	
MTP-047	7.7	0%	EGFR G719X		WT		Cobas detected more mutations	Cobas detected more mutations	EGFR G719A	
MTP-048	2.7	61%	EGFR exon19 del		EGFR exon19 del p.L747_P753>S		Concordant	Concordant	EGFR exon19 del	EGFR T790M
MTP-049	8.4	0%	EGFR exon19 del		EGFR exon19 del p.L747_A750>P		Concordant	Concordant	EGFR exon19 del	
MTP-050	5.8	0%	EGFR		EGFR exon19 del	KRAS	Concordant	USK detected	EGFR	

			exon19 del		p.L747_P753>S	p.Q61H*			more mutations	exon19 del
MTP-051	6.4	0%	EGFR L858R		EGFR L858R			Concordant	Concordant	EGFR L858R
MTP-052	9.4	0%	EGFR exon19 del		EGFR exon19 del E746_A750del			Concordant	Concordant	EGFR exon19 del
MTP-053	N/A	N/A	EGFR L858R	EGFR T790M	EGFR L858R	EGFR T790M	BRAF p.V600E*	Concordant	USK detected more mutations	EGFR L858R
MTP-054	4.4	0%	EGFR exon19 del		Exon19 del p.E746_S752>V	Exon19 del p.L747_A750>P		USK detected more mutations	USK detected more mutations	N/A
MTP-055	N/A	N/A	EGFR L858R		EGFR L858R			Concordant	Concordant	EGFR L858R
MTP-056	N/A	N/A	EGFR exon19 del	EGFR T790M	EGFR exon19 del p.L747_A750>P	EGFR T790M		Concordant	Concordant	N/A
MTP-057	N/A	N/A	EGFR exon19 del	EGFR T790M	EGFR exon19 del p.L747_P753>S	EGFR T790M		Concordant	Concordant	N/A
MTP-058	N/A	N/A	EGFR exon19 del		EGFR exon19 del p.L747_A750>P	KRAS p.G12R/C*		Concordant	USK detected more mutations	EGFR exon19 del
MTP-059	6.1	4%	EGFR exon19 del		WT			Cobas detected more mutations	Cobas detected more mutations	EGFR exon19 del
MTP-060	20.3	0%	EGFR G719X		EGFR G719A			Concordant	Concordant	N/A
MTP-061	11.3	0%	WT		KRAS p.G12D*			Concordant	USK detected more mutations	N/A
MTP-062	3.3	2%	WT		WT			Concordant	Concordant	N/A
MTP-063	N/A	N/A	WT		WT			Concordant	Concordant	WT
MTP-064	19.7	2%	WT		KRAS p.G13C*			Concordant	USK detected more mutations	N/A
MTP-065	N/A	N/A	WT		KRAS p.Q61H*			Concordant	USK detected more mutations	N/A
MTP-066	N/A	N/A	WT		WT			Concordant	Concordant	N/A
MTP-067	N/A	N/A	WT		WT			Concordant	Concordant	N/A
MTP-068	N/A	N/A	WT		WT			Concordant	Concordant	N/A
MTP-069	9.0	0%	WT		WT			Concordant	Concordant	N/A
MTP-070	5.2	0%	WT		WT			Concordant	Concordant	N/A
MTP-071	N/A	N/A	WT		WT			Concordant	Concordant	N/A

MTP-072	N/A	N/A	WT	KRAS p.G12A/V*	BRAF p.V600E*	Concordant	USK detected more mutations	N/A
MTP-073	4.0	0%	WT	WT		Concordant	Concordant	EGFR exon19 del
MTP-074	2.7	0%	WT	WT		Concordant	Concordant	N/A
MTP-075	N/A	N/A	WT	WT		Concordant	Concordant	N/A
MTP-076	N/A	N/A	WT	WT		Concordant	Concordant	EGFR exon19 del
MTP-077	6.4	52%	WT	WT		Concordant	Concordant	N/A
MTP-078	12.0	74%	WT	WT		Concordant	Concordant	EGFR L858R
MTP-079	N/A	N/A	WT	WT		Concordant	Concordant	EGFR exon19 del
MTP-080	N/A	N/A	WT	WT		Concordant	Concordant	N/A
MTP-081	N/A	N/A	WT	EGFR L858R		USK detected more mutations	USK detected more mutations	EGFR L858R
MTP-082	3.5	0%	WT	WT		Concordant	Concordant	N/A
MTP-083	8.5	0%	WT	WT		Concordant	Concordant	N/A
MTP-084	15.4	1%	WT	KRAS p.G12C*		Concordant	USK detected more mutations	N/A
MTP-085	N/A	N/A	WT	WT		Concordant	Concordant	N/A
MTP-086	24.6	0%	WT	KRAS p.Q61H*		Concordant	USK detected more mutations	N/A
MTP-087	3.2	0%	WT	WT		Concordant	Concordant	N/A
MTP-088	5.1	1%	WT	EGFR exon20 ins p.D770_N771ins G		USK detected more mutations	USK detected more mutations	N/A
MTP-089	3.1	4%	WT	WT		Concordant	Concordant	N/A
MTP-090	N/A	N/A	WT	EGFR exon19 del p.L747_A750>P		USK detected more mutations	USK detected more mutations	EGFR exon19 del
MTP-091	2.8	0%	WT	WT		Concordant	Concordant	N/A
MTP-092	12.8	0%	WT	WT		Concordant	Concordant	N/A
MTP-093	3.5	2%	WT	WT		Concordant	Concordant	N/A
MTP-094	5.7	0%	WT	KRAS p.G12S*	BRAF p.V600E*	Concordant	USK detected more mutations	N/A
MTP-095	6.9	0%	WT	WT		Concordant	Concordant	N/A
MTP-096	3.5	13%	WT	WT		Concordant	Concordant	N/A
MTP-097	6.6	26%	WT	WT		Concordant	Concordant	N/A

MTP-098	N/A	0%	WT		EGFR G719A	EGFR L861Q/R*		USK detected more mutations	USK detected more mutations	EGFR G719A	EGFR L861Q
MTP-099	2.8	0%	WT		WT			Concordant	Concordant	N/A	
MTP-100	N/A	0%	WT		WT			Concordant	Concordant	N/A	
GRO-01	7.8	0%	WT		EGFR L858R	EGFR T790M		USK detected more mutations	USK detected more mutations	EGFR L858R	EGFR T790M
GRO-02	10	7%	WT		KRAS p.G12D*			Concordant	USK detected more mutations	N/A	
GRO-03	10	0%	WT		WT			Concordant	Concordant	N/A	
GRO-04	2.8	0%	EGFR exon19 del		EGFR exon19 del p.L747_A750>P			Concordant	Concordant	N/A	
GRO-05	10	20%	WT		WT			Concordant	Concordant	N/A	
GRO-06	4.5	3%	EGFR L858R		EGFR L858R			Concordant	Concordant	EGFR L858R	
GRO-07	10	5%	EGFR exon19 del		EGFR exon19 del p.E746_A750del			Concordant	Concordant	EGFR exon19 del	
GRO-08	7.5	3%	EGFR exon19 del		EGFR exon19 del p.L747_A750>p			Concordant	Concordant	EGFR exon19 del	
GRO-09	2.9	3%	WT		WT			Concordant	Concordant	N/A	
GRO-10	2.3	0%	EGFR L858R		EGFR L858R			Concordant	Concordant	EGFR L858R	
GRO-11	10	2%	EGFR exon19 del	EGFR T790M	EGFR exon19 del p.L747_A750>P	EGFR T790M		Concordant	Concordant	EGFR exon19 del	EGFR T790M
GRO-12	3.9	0%	WT		WT			Concordant	Concordant	N/A	
GRO-13	10	0%	EGFR L858R		EGFR L858R			Concordant	Concordant	EGFR L858R	
GRO-14	5.1	0%	EGFR exon19 del	EGFR T790M	EGFR exon19 del p.E746_A750del	EGFR T790M		Concordant	Concordant	EGFR exon19 del	EGFR T790M
GRO-15	8.5	0%	EGFR exon19 del		EGFR exon19 del p.L747_A750>P			Concordant	Concordant	EGFR exon19 del	
GRO-16	8.6	7%	EGFR exon19 del	EGFR T790M	EGFR exon19 del p.L747_P753>S	EGFR T790M		Concordant	Concordant	EGFR exon19 del	EGFR T790M
GRO-17	4.8	0%	EGFR G719X		EGFR G719C	KRAS p.G12R/C*	PIK3CA p.E542K*	Concordant	USK detected more mutations	EGFR G719A	
GRO-18	10.1	3%	EGFR exon19 del	EGFR T790M	EGFR exon19 del p.L747_P753>S	EGFR T790M		Concordant	Concordant	EGFR exon19 del	EGFR T790M
GRO-19	10	1%	WT		WT			Concordant	Concordant	N/A	
GRO-20	10	1%	WT		KRAS p.G12A/V*			Concordant	USK detected more mutations	N/A	

GRO-21	10	0%	WT		WT			Concordant	Concordant	EGFR G719A	EGFR E709A	
GRO-22	6.9	10%	EGFR exon19 del		EGFR exon19 del p.L747_P753>S			Concordant	Concordant	EGFR exon19 del		
GRO-23	9.9	0%	EGFR exon19 del		EGFR exon19 del p.E746_A750del	EGFR T790M		USK detected more mutations	USK detected more mutations	EGFR exon19 del		
GRO-24	2.8	0%	WT		WT			Concordant	Concordant	N/A		
GRO-25	6.5	44%	WT		WT			Concordant	Concordant	N/A		
GRO-26	10	19%	EGFR L858R		WT			Cobas detected more mutations	Cobas detected more mutations	EGFR L858R		
GRO-27	10	0%	EGFR exon19 del	EGFR T790M	EGFR exon19 del p.E746_S752>V	EGFR T790M		Concordant	Concordant	EGFR exon19 del	EGFR T790M	
GRO-28	7.3	0%	EGFR L858R		WT			Cobas detected more mutations	Cobas detected more mutations	EGFR L858R		
GRO-29	10.1	0%	EGFR L858R	EGFR T790M	EGFR L858R	EGFR T790M		Concordant	Concordant	EGFR L858R	EGFR T790M	
GRO-30	10	1%	EGFR L858R	EGFR T790M	EGFR L858R	EGFR T790M		Concordant	Concordant	EGFR L858R		
GRO-31	10	0%	EGFR exon19 del	EGFR T790M	EGFR exon19 del p.E746_A750del	EGFR T790M		Concordant	Concordant	EGFR exon19 del	EGFR T790M	
GRO-32	3.9	0%	EGFR exon19 del†		WT			Concordant	Cobas detected more mutations	EGFR exon19 del		
GRO-33	N/A	N/A	EGFR L858R	EGFR T790M	EGFR L858R	EGFR T790M	PIK3CA p.E542K*	Concordant	USK detected more mutations	EGFR L858R		
GRO-34	9.3	7%	EGFR exon19 del	EGFR T790M	Exon19 del p.E746_A750del			Cobas detected more mutations	Cobas detected more mutations	EGFR exon19 del		
GRO-35	9.5	19%	EGFR exon19 del	EGFR T790M	Exon19 del p.E746_A750del	EGFR T790M		Concordant	Concordant	EGFR exon19 del	EGFR T790M	
GRO-36	10	2%	EGFR L858R	EGFR T790M	EGFR L858R	EGFR T790M	EGFR C797S*	Concordant	USK detected more mutations	EGFR L858R	EGFR T790M	EGFR C797S
GRO-37	7.9	14%	EGFR exon19 del	EGFR T790M	Exon19 del p.E746_A750del	EGFR T790M		Concordant	Concordant	EGFR exon19 del	EGFR T790M	

*Mutation not detectable on Cobas panel, †Mutation not detectable on UltraSEEK panel, USK: UltraSEEK, N/A: not available.

Table S3. Calculations of agreement using Cohen's κ.

**MTP and GRO Cohorts**			
**Mutations Detectable on Both Panels**		**UltraSEEK**	
		Detected	Not Detected
Cobas	Detected	71	10
	Not Detected	9	47
	Pe	0.52	
	Cohen's κ	0.71	
**All Clinically Relative Mutations**		**UltraSEEK**	
		Detected	Not Detected
Cobas	Detected	64	11
	Not Detected	25	57
	Pe	0.50	
	Cohen's κ	0.54	
**Harmonized Input Cohort**			
**Mutations Detectable on Both Panels**		**UltraSEEK**	
		Detected	Not Detected
Cobas	Detected	22	1
	Not Detected	9	27
	Pe	0.49	
	Cohen's κ	0.66	
**All Clinically Relative Mutations**		**UltraSEEK**	
		Detected	Not Detected
Cobas	Detected	22	1
	Not Detected	15	21
	Pe	0.47	
	Cohen's κ	0.49	

Table S4. Concordance of mutation detection between UltraSEEK™ and Cobas® according to ccfDNA input amount excluding mutation-negative plasma samples.

<table>
<tr><th>Input*</th><th>Cases</th><th>Concordant</th><th>Cobas Detected More Mutations</th><th>UltraSEEK Detected More Mutations</th><th>Grouped Concordance</th></tr>
<tr><td>Negative†</td><td>12</td><td>8 (67%)</td><td>1 (8%)</td><td>3 (25%)</td><td>8 (67%)</td></tr>
<tr><td>2–5 ng</td><td>19</td><td>16 (84%)</td><td>2 (11%)</td><td>1 (5%)</td><td rowspan="2">27 (69%)</td></tr>
<tr><td>5–8 ng</td><td>20</td><td>11 (55%)</td><td>5 (25%)</td><td>4 (20%)</td></tr>
<tr><td>8–10 ng</td><td>23</td><td>20 (87%)</td><td>2 (9%)</td><td>1 (4%)</td><td rowspan="2">37 (93%)</td></tr>
<tr><td>>10 ng</td><td>17</td><td>17 (100%)</td><td>0 (0%)</td><td>0 (0%)</td></tr>
</table>

*as determined by LiquidIQ® analysis. †Level of amplifiable copies too low for accurate calling and considered unevaluable.

Table S5. Concordance of detected mutations between UltraSEEK™ and the diagnostic Cobas® test with harmonized input.

Sample	LiquidIQ Results		Cobas Mutations		UltraSEEK Mutations			Concordance		EGFR Tissue Mutations		
	Input (ng)	WBC Contamination	Mutation 1	Mutation 2	Mutation 1	Mutation 2	Mutation 3	Detectable on Both Panels	Clinically Relevant Mutations	Mutation 1	Mutation 2	Mutation 3
MTP-001	2.8	0%	WT		EGFR exon19 del p.L474_P753>S			USK detected more mutations	USK detected more mutations	EGFR exon19 del		
MTP-002	13.2	0%	EGFR L858R		EGFR L858R			Concordant	Concordant		N/A	
MTP-003	10.8	0%	EGFR L858R		EGFR L858R			Concordant	Concordant	EGFR L858R		
MTP-004	9.6	0%	EGFR L858R		EGFR L858R			Concordant	Concordant		N/A	
MTP-005	3.1	0%	WT		EGFR G719A	EGFR L861Q/R*		USK detected more mutations	USK detected more mutations	EGFR G719X	EGFR L861R	
MTP-006	4.9	0%	EGFR L858R		EGFR L858R			Concordant	Concordant		N/A	
MTP-007	9.3	0%	EGFR exon19 del		EGFR exon19 del p.L474_A750>P			Concordant	Concordant	EGFR exon19 del		
MTP-008	24.4	10%	EGFR L858R		EGFR L858R			Concordant	Concordant		N/A	
MTP-009	5.6	0%	WT		WT			Concordant	Concordant		N/A	
MTP-010	4.8	0%	EGFR exon19 del		EGFR exon19 del E746_A750del			Concordant	Concordant	EGFR exon19 del		
MTP-011	9.8	0%	EGFR L858R		EGFR L858R			Concordant	Concordant	WT		
MTP-012	6.9	0%	EGFR L858R		EGFR L858R			Concordant	Concordant		N/A	
MTP-013	3.0	6%	WT		WT			Concordant	Concordant	EGFR exon19 del		
MTP-014	12.2	6%	EGFR L858R		EGFR L858R			Concordant	Concordant	WT		
MTP-015	9.6	0%	EGFR exon19 del		EGFR exon19 del E746_A750del			Concordant	Concordant		N/A	
MTP-016	N/A	N/A	WT		EGFR exon19 del E746_A750del			USK detected more mutations	USK detected more mutations	EGFR exon19 del		
MTP-017	10.8	0%	EGFR exon19 del		EGFR exon19 del E746_A750del			Concordant	Concordant		N/A	
MTP-018	14.3	0%	EGFR exon19 del	EGFR T790M	EGFR exon19 del E746_A750del	EGFR T790M		Concordant	Concordant		N/A	

MTP-019	6.8	0%	EGFR exon19 del		EGFR exon19 del E746_A750del	EGFR T790M	USK detected more mutations	USK detected more mutations	EGFR exon19 del	
MTP-020	9.2	0%	EGFR exon19 del	EGFR T790M	EGFR exon19 del p.E746_S752>V	EGFR T790M	Concordant	Concordant	EGFR exon19 del	
MTP-021	6.1	0%	WT		WT		Concordant	Concordant	EGFR exon19 del	
MTP-022	6.2	0%	EGFR exon19 del		EGFR exon19 del p.E746_S752>V	EGFR T790M	USK detected more mutations	USK detected more mutations	EGFR exon19 del	
MTP-023	6.6	0%	EGFR L858R	EGFR T790M	EGFR L858R	EGFR T790M	Concordant	Concordant	EGFR L858R	
MTP-024	4.9	0%	EGFR exon19 del		EGFR exon19 del p.L747_A750>P		Concordant	Concordant	EGFR exon19 del	
MTP-025	11.5	0%	EGFR L858R		EGFR L858R		Concordant	Concordant	EGFR L858R	
MTP-026	14.0	3%	EGFR exon19 del	EGFR T790M	EGFR exon19 del E746_A750del	EGFR T790M	Concordant	Concordant	EGFR exon19 del	
MTP-027	4.9	0%	EGFR G719X	EGFR S768I	EGFR G719C	EGFR S768I	Concordant	Concordant	EGFR G719C	EGFR S768I
MTP-028	6.1	0%	WT		EGFR exon19 del p.E746_S752>V	EGFR T790M	USK detected more mutations	USK detected more mutations	EGFR exon19 del	
MTP-029	3.1	0%	WT		WT		Concordant	Concordant	EGFR exon19 del	
MTP-030	22.7	32%	EGFR L861Q	EGFR T790M	EGFR L861Q	EGFR T790M	Concordant	Concordant	EGFR L861Q	
MTP-031	13.1	0%	EGFR exon19 del	EGFR T790M	EGFR exon19 del E746_A750del	EGFR T790M	Concordant	Concordant	EGFR exon19 del	
MTP-032	11.6	0%	EGFR exon19 del	EGFR T790M	EGFR exon19 del E746_A750del	EGFR T790M	Concordant	Concordant	EGFR exon19 del	
MTP-061	11.3	0%	WT		KRAS p.G12D*		Concordant	USK detected more mutations	N/A	
MTP-062	3.3	2%	WT		WT		Concordant	Concordant	N/A	
MTP-063	N/A	N/A	Invalid		WT		Concordant	Concordant	WT	
MTP-064	19.7	2%	WT		KRAS p.G13C*		Concordant	USK detected more mutations	N/A	
MTP-065	N/A	N/A	WT		KRAS p.Q61H*		Concordant	USK detected more mutations	N/A	
MTP-066	N/A	N/A	WT		WT		Concordant	Concordant	N/A	
MTP-067	N/A	N/A	Invalid		WT		Concordant	Concordant	N/A	
MTP-068	N/A	N/A	Invalid		WT		Concordant	Concordant	N/A	
MTP-069	9.0	0%	WT		WT		Concordant	Concordant	N/A	
MTP-070	5.2	0%	WT		WT		Concordant	Concordant	N/A	

MTP-071	N/A	N/A	WT	WT		Concordant	Concordant	N/A	
MTP-072	N/A	N/A	WT	KRAS p.G12A/V*	BRAF p.V600E*	Concordant	USK detected more mutations	N/A	
MTP-073	4.0	0%	WT	WT		Concordant	Concordant	EGFR exon19 del	
MTP-074	2.7	0%	WT	WT		Concordant	Concordant	N/A	
MTP-075	N/A	N/A	EGFR exon19 del	WT		Cobas detected more mutations	Cobas detected more mutations	N/A	
MTP-076	N/A	N/A	WT	WT		Concordant	Concordant	EGFR exon19 del	
MTP-077	6.4	52%	WT	WT		Concordant	Concordant	N/A	
MTP-078	12.0	74%	WT	WT		Concordant	Concordant	EGFR L858R	
MTP-079	N/A	N/A	Invalid	WT		Concordant	Concordant	EGFR exon19 del	
MTP-080	N/A	N/A	WT	WT		Concordant	Concordant	N/A	
MTP-081	N/A	N/A	WT	EGFR L858R		USK detected more mutations	USK detected more mutations	EGFR L858R	
MTP-082	3.5	0%	WT	WT		Concordant	Concordant	N/A	
MTP-083	8.5	0%	WT	WT		Concordant	Concordant	N/A	
MTP-084	15.4	1%	WT	KRAS p.G12C*		Concordant	USK detected more mutations	N/A	
MTP-085	N/A	N/A	WT	WT		Concordant	Concordant	N/A	
MTP-086	24.6	0%	WT	KRAS p.Q61H*		Concordant	USK detected more mutations	N/A	
MTP-087	3.2	0%	WT	WT		Concordant	Concordant	N/A	
MTP-088	5.1	1%	WT	EGFR exon20 ins p.D770_N771insG		USK detected more mutations	USK detected more mutations	N/A	
MTP-089	3.1	4%	WT	WT		Concordant	Concordant	N/A	
MTP-090	N/A	N/A	WT	EGFR exon19 del p.L747_A750>P		USK detected more mutations	USK detected more mutations	EGFR exon19 del	
MTP-091	2.8	0%	WT	WT		Concordant	Concordant	N/A	

*Mutation not detectable on Cobas panel, †Mutation not detectable on UltraSEEK panel, USK: UltraSEEK, N/A: not available.

Table S6. Concordance of mutation detection related to the harmonized ccfDNA input excluding mutation-negative plasma samples.

Input*	Cases	Concordant	Cobas Detected More Mutations	UltraSEEK Detected More Mutations	Grouped Concordance
Negative†	4	0 (0%)	1 (25%)	3 (75%)	0 (0%)
2–5 ng	6	4 (67%)	0 (0%)	2 (33%)	7 (54%)
5–8 ng	7	3 (43%)	0 (0%)	4 (57%)	
8–10 ng	5	5 (100%)	0 (0%)	0 (0%)	16 (100%)
>10 ng	11	11 (100%)	0 (0%)	0 (0%)	

Data is excluding the four cases of which the Cobas® test was invalid. *as determined by LiquidIQ® analysis. †Level of amplifiable copies too low for accurate calling and considered negative.

© 2020 by the authors. Licensee MDPI, Basel, Switzerland. This article is an open access article distributed under the terms and conditions of the Creative Commons Attribution (CC BY) license (http://creativecommons.org/licenses/by/4.0/).
